# Dual-use capabilities of concern of biological AI models

**DOI:** 10.1371/journal.pcbi.1012975

**Published:** 2025-05-08

**Authors:** Jaspreet Pannu, Doni Bloomfield, Robert MacKnight, Moritz S. Hanke, Alex Zhu, Gabe Gomes, Anita Cicero, Thomas V. Inglesby

**Affiliations:** 1 Center for Health Security, Bloomberg School of Public Health, Johns Hopkins University, Baltimore, Maryland,; 2 Department of Health Policy, Stanford School of Medicine, Stanford University, Stanford, California, United States of America; 3 School of Law, Fordham University, New York, New York, United States of America; 4 Department of Chemical Engineering, Carnegie Mellon University, Pittsburg, Pennsylvania, United States of America; 5 Department of Chemistry, Carnegie Mellon University, Pittsburg, Pennsylvania, United States of America; 6 Wilton E. Scott Institute for Energy Innovation, Carnegie Mellon University, Pittsburg, Pennsylvania, United States of America; University of Virginia, UNITED STATES OF AMERICA

## Abstract

As a result of rapidly accelerating artificial intelligence (AI) capabilities, multiple national governments and multinational bodies have launched efforts to address safety, security and ethics issues related to AI models. One high priority among these efforts is the mitigation of misuse of AI models, such as for the development of chemical, biological, nuclear or radiological (CBRN) threats. Many biologists have for decades sought to reduce the risks of scientific research that could lead, through accident or misuse, to high-consequence disease outbreaks. Scientists have carefully considered what types of life sciences research have the potential for both benefit and risk (dual use), especially as scientific advances have accelerated our ability to engineer organisms. Here we describe how previous experience and study by scientists and policy professionals of dual-use research in the life sciences can inform dual-use capabilities of AI models trained using biological data. Of these dual-use capabilities, we argue that AI model evaluations should prioritize addressing those which enable high-consequence risks (i.e., large-scale harm to the public, such as transmissible disease outbreaks that could develop into pandemics), and that these risks should be evaluated prior to model deployment so as to allow potential biosafety and/or biosecurity measures. While biological research is on balance immensely beneficial, it is well recognized that some biological information or technologies could be intentionally or inadvertently misused to cause consequential harm to the public. AI-enabled life sciences research is no different. Scientists’ historical experience with identifying and mitigating dual-use biological risks can thus help inform new approaches to evaluating biological AI models. Identifying which AI capabilities pose the greatest biosecurity and biosafety concerns is necessary in order to establish targeted AI safety evaluation methods, secure these tools against accident and misuse, and avoid impeding immense potential benefits.

## Introduction

Scientists can perform almost all biological research in ways that pose minimal risk to society. However, some limited areas of life sciences research can subject the public to risks from high-consequence pathogens, through laboratory accidents or misuse. These risks may be exacerbated by our ability to manipulate and engineer increasingly complex biomolecules and biomolecular systems. Researchers can now also combine rapidly improving artificial intelligence (AI) models with wet-lab advances to facilitate, accelerate and augment this work (see Box 1).

Within the biological sciences, AI models can make use of and generate biological information in the form of images, genetic sequences, protein sequences, protein structure, or even more sophisticated biological complexes. Biological AI models—models trained on or capable of meaningfully manipulating substantial quantities of biological data [1]—have already surpassed human performance on multiple tasks, and their development is advancing rapidly [2]. While prior efforts to collect biological sequence and structure data have been successful, functional biological data remains a barrier to capability advancement, as well as appropriate data curation and annotation for AI applications.

As with all tools that allow us to better manipulate biology, future advanced AI models have the potential to be misused or misapplied, and these biosecurity risks have been publicly noted by scientists and model developers. Baker and Church note that AI protein design models are “vulnerable to misuse and the production of dangerous biological agents” while Boiko and colleagues include a security supplement in which they state that they “strongly believe that guardrails must be put in place to prevent … dual use of large language models” for autonomous completion of chemical and biological synthesis protocols [3,4]. Two authors of this paper were co-authors on Boiko and colleagues [4].

**Box 1.– Recent biosecurity-relevant trends in AI development**

Concerns regarding potential biosecurity risks have been bolstered by recent trends in AI development.

1. *Large-language models* (LLMs) such as GPT-4 and 4o have shown rapid progress in dual-use capabilities, including assisting with completing biological and chemical research design and testing.
2. AI systems which include biological information, data and outputs, which we term *biological AI models* [1], have seen a similar rate of progress and model size expansion [2]. This progress suggests the possibility that *biological foundation models* could be built: large, general-purpose models that capture the complexity of biology.
3. Advances in LLMs and biological AI models are complementary. There are initial indications that LLMs can assist users in accessing biological AI models to perform complex scientific tasks. These advances are in the future likely to lower the cost of achieving biological breakthroughs and allow less experienced researchers to use increasingly complex and powerful biological tools.
4. LLMs, biological AI models, and integrations of the two, can also interface with *AI-enabled autonomous laboratory environments* [4]. These capabilities further reduce the time, expertise, and equipment required to synthesize pathogens, and suggest the possibility of end-to-end or “full stack” AI tool development in this domain.

In considering the biosecurity and biosafety implications of biological AI models, it is important to consider the extent to which a model contributes to risks beyond preexisting technologies such as internet search engines [5]. AI model developers and policymakers have not yet broadly agreed upon what model features or uses most increase significant biosecurity and biosafety risks to the public—or what forms of risks are most worth mitigating. As a result, the limited published biosecurity studies of AI models done to date mainly assess LLMs [6,7], and test for different risks using differing assumptions regarding which threats should be guarded against. The limited number and scope of these studies make it challenging to use them to predict the capabilities and risk profiles of AI models released in the coming months and years. Empirical observations by AI developers indicate that AI model capabilities will continue to exponentially progress [8] contingent on data availability.

None of the small studies in the field so far have assessed how foundation models specifically trained on relevant biological data will marginally increase risks to the public, nor have they assessed how the integration or “stacking” of different types of AI tools (e.g. LLMs, biological AI models, and autonomous robotics) change those risks [9]. Integrations that combine AI tools for biological research are increasingly common. For example, there are efforts to enable access to leading open-source protein and molecular design tools using directly integrated AI chatbot interfaces, also termed “copilots” [10]. Researchers and companies are seeking to develop autonomous or semi-autonomous AI agents capable of conducting science [4,11]. Some AI tools provide assistance to novices in completing complex research tasks (termed AI “uplift”) [6], while others aim to eliminate humans-in-the-loop entirely [12]. To date, there are no published third-party biosecurity assessments of AI models trained on biological sequence and/or structure data (as compared to LLMs), though more than 100 individuals, many of whom are academic developers of such models, have signed voluntary commitments to conduct “evaluations of AI systems to identify meaningful safety and security concerns prior to release” and to engage “in the joint development of evaluation frameworks” [13]. Two authors of this paper signed this statement in support.

A current barrier to increased model capability includes functional biological data (as compared to sequence or static structural data). However, a growing proportion of wet-lab work for data generation, such as data visualization, data analysis, and sample creation, can be facilitated by autonomous machines increasingly enabled by AI, including machines that researchers pay to access remotely, known as cloud labs [14–16]. Foundation models, even those untrained for this purpose, have shown facility at directing robots to perform laboratory tasks [17]. Skilled AI agents that engage in sophisticated planning and operations work also enable this work [12,18]. Taken together, these facts suggest that AI model capabilities may play an increasingly large role in enabling high-consequence biosecurity risks in the coming years.

### Policymaker guidance for hazardous biological AI capabilities to date

In part to address these concerns, the governments of the United States and United Kingdom are working with scientists and model developers to take new steps toward designing AI biosecurity evaluations [19]. *Evaluations* in this context refer to techniques for assessing an AI models’ capabilities, especially capabilities that can cause harm [20]. AI evaluations to date have included automated tasks (e.g., multiple choice questions), dynamic studies in which humans or other AI models attempt to elicit harmful capabilities (“red-teaming”), and randomized trials in which individuals or groups are set to a task with or without access to an AI model (“human uplift studies”), among other methods [20].

Neither the UK nor US governments have yet released standardized biosecurity evaluation methods, though both have indicated their intention to do so. In September 2023, the UK government was the first to establish an expert-led taskforce to address risks from frontier AI models [21]. Subsequently in November 2023, they launched the UK AI Safety Institute, which is responsible for creating and conducting evaluations on leading AI models. The Institute is especially focused on “containing risks that pose significant large-scale harm if left unchecked: chemical and biological capabilities, and cyber offense capabilities” [22]. It has recently released an open-source framework for LLM evaluations called Inspect [23]. The US and UK (alongside several other nations) also signed the Bletchley declaration in November 2023, acknowledging the potential risks of AI [24].

Concurrently in October 2023, the White House promulgated the Executive Order on the Safe, Secure, and Trustworthy Development and Use of Artificial Intelligence (2023 AI EO) [25]. The 2023 AI EO directed various federal agencies with creating “robust, reliable, repeatable and standardized evaluations of AI systems,” with a planned focus on evaluating hazardous biological AI model capabilities among a small number of other significant risks. A subsequent National Security Memorandum published in October 2024 further directed the Department of Defense to “advance classified evaluations of advanced AI models’ capacity to generate or exacerbate deliberate (…) biological threats” [26]. Of note, the 2023 AI EO focused on foundation models—extremely large AI models trained on broad data, containing at least tens of billions of parameters, and exhibiting high levels of performance on various tasks. Of particular concern was “substantially lowering the barrier of entry for non-experts to design, synthesize, acquire, or use chemical, biological, radiological or nuclear (CBRN) weapons”. The 2023 AI EO also called for investing in the use of AI to mitigate such threats. With regards to foundation models trained on primarily biological sequence data, those models which use greater than 10^23^ FLOPS of computational power were required to be reported to the US Department of Commerce. Since then, genomic foundation models exceeding this threshold have been created [27,28].

Under the 2023 AI EO, the US National Institute of Standards and Technology (NIST) launched the US AI Safety Institute, which is charged with proposing “guidance and benchmarks for evaluating and auditing AI capabilities with a focus on capabilities through which AI could cause harm,” including by biological means [29]. In May 2024 the US Office of Science and Technology Policy also recommended oversight of dual-use computational models that could enable the design of novel biological agents or enhanced pandemic pathogens [30], given the high-consequence risks these agents and pathogens could pose to the public. In January 2025, the incoming US administration announced the revocation of the 2023 AI EO and thus the removal of reporting requirements for models surpassing the above computational thresholds [31], however, the US AI Safety Institute remains in place.

Additional governmental bodies are continuing to become involved in AI governance. In 2024, the European Union launched an AI Office to monitor, supervise and enforce the EU AI Act, which governs general-purpose models with systemic risk, though models used exclusively for scientific research are excluded from oversight [32].

## Proposed approach to determining biological AI capabilities of concern

### Prior experience studying dual-use life sciences research can inform AI capabilities of concern

There is no common AI industry approach to evaluating biological AI models for risks. There is, however, prior guidance from scientists, public health professionals, and policymakers regarding life sciences research that “could be directly misapplied to pose a significant threat with broad potential consequences to public health and safety,” known as dual-use research of concern [33]. There is also guidance regarding life sciences research that could result in “enhanced potential pandemic pathogens” [34]. A recent 2024 update to these US policies incorporates lessons learned from prior attempts to govern this realm of research, makes substantial improvements, and includes recommendations to address risks from computational approaches in this domain [30].

The AI model biosecurity conundrum—how to retain AI’s significant benefits while heading off serious concerns around AI model misuse—finds a ready parallel with dual-use research of concern and research intended to create enhanced potential pandemic pathogens. Scientists and policymakers have spent years analyzing which forms of life-sciences research pose serious risks through accidental release, or inadvertent or deliberate misuse [33,35–38]. Although these recommendations have historically been targeted at wet-lab experimentation, they have also been used to assess what information should be shared publicly [33,39,40], also known as information hazards [41]—the type of concern that is critical to consider for AI models with biological information and capabilities. As the AI community develops evaluations, they should take advantage of the scientific expertise and governmental experience instantiated in prior dual-use study, along with incorporating improvements based on how these prior recommendations were insufficient.

Fundamentally, biological AI models aim to greatly facilitate complex wet-lab work, or do entirely in silico that which can only be done now in vitro or in vivo—and in doing so, make it easier for those with access to a relevant model to reduce or dispense with time-consuming and expensive steps. Because biological systems are complex, traditional dual-use research (e.g., studies analyzing pathogen features such as tropism, transmissibility, and virulence) has historically relied on trial and error, methodical experimentation, or directed evolution [36]. AI may allow users to conduct this research faster and at lower cost, as it has in other related research domains [42–46].

### Evaluations should assess capabilities, not specific pathogens or threat scenarios

Many biosafety and biosecurity governance approaches have in the past relied on taxonomic lists of specific pathogens to be regulated [47–49]. However, we recommend that evaluation approaches instead focus on AI-enabled *capabilities*, rather than AI engagement with risks related to specific pathogens. When applying biosecurity in practice, pathogen lists are “both too specific and too ambiguous for many of the uses to which they are applied” [50]. Experts have recognized the shortcomings of pathogen lists for over a decade, and have instead emphasized oversight based on the intention of the research being conducted (for example, an intention to increase a pathogen’s transmissibility) [51]. Many National Academies assessments [35,36,52] seek to avoid taxonomic pathogen lists, given the modular nature of modern biotechnology, which increasingly makes use of parts of organisms to confer new traits. One influential report championed “adopting a broader perspective on the threat spectrum” and urged policymakers to recognize “the limitations inherent in any agent-specific threat list.” Biosecurity measures, the authors argued, should focus instead on the “intrinsic properties of pathogens and toxins that render them a threat, and how such properties … could be manipulated by evolving technologies” [52]. The U.S. National Science Advisory Board for Biosecurity, as well, in 2024 underlined the importance of reviewing experiments for dual-use potential by analyzing whether the experiment involves risky pathogenic characteristics rather than whether it involves specific pathogens and experimental methods [53]. The recently released United States Government Policy for Oversight of Dual Use Research of Concern and Pathogens with Enhanced Pandemic Potential also places highest priority focus (Category 2) on experiments capable of enhancing the pandemic potential of a pathogen “such that it may pose a significant threat to public health” regardless of the specific progenitor pathogen [30].

When it comes to AI evaluations, too, this capabilities-based approach should be pursued as compared to a list-based approach. Although pathogen list-based approaches create bright lines that make policies easy to follow, they also lack the flexibility to account for emerging technology developments. Such lists can also provide an unwarranted sense of security, reducing vigilance and surveillance of the technological horizon for newly emerging capabilities and serious risks [52]. Taxonomic lists remain useful tools for controlling access to whole organisms, such as for law enforcement or via export controls [50]. However, in the setting of AI where novel risks must be anticipated, capability assessments and anticipating high-consequence harms to the public, not specified pathogens, are better suited to inform risk assessments. As the National Academies panel co-chaired by Stanley Lemon and David Relman (the Lemon-Relman Report) concluded, it is futile to predict how exactly future terrorists or malicious states will attempt to misuse biology [52].

### Prioritize evaluations of biological capabilities that enable high-consequence harms

As governments and AI developers design capability-based AI model biosecurity evaluations, they should focus first on capabilities which enable high-consequence harms to the public: that is, those capabilities which could enable, accelerate, or simplify the creation of transmissible biological constructs that could lead to human pandemics, or similar pandemic-like events in animals, plants or the environment.

It will be a significant challenge to evaluate AI models for their ability to contribute to any possible biology-related accident or misdeed, no matter how limited the consequences. Given the effort required, evaluations should first prioritize assessing the highest consequence harms to the public, such as pandemics. We recognize this prioritization differs from traditional biosafety practices, which give greater attention to less consequential, but more common and expected risks. However, we believe prioritization of high consequence is warranted given the large-scale harms to the public that can result from transmissible, pandemic-capable pathogens, as has been noted by other researchers [54]. This prioritization of pandemic-capable pathogens is also reflected in the United States Government Policy for Oversight of Dual Use Research of Concern and Pathogens with Enhanced Pandemic Potential, where highest priority for oversight is given to Category 2 research involving potential pandemic pathogens [30]. Non-transmissible pathogens and toxins, while also potentially dangerous to individuals and even large groups of people, are self-limited. In contrast, the unchecked spread of transmissible, pandemic-capable pathogens can strain health systems and require severe containment measures which also contribute to public harm; therefore, prevention should be prioritized.

Such prioritizing within biological AI model evaluations would, if implemented correctly, help to limit widespread access to the most concerning models, or require model safety modifications before deployment for others. As experts have long recognized, attempts to constrain the flow of scientific information, especially in biology, face acute practical and legal challenges [35,52]. It is thus all the more important for biosecurity evaluations and responses in the AI setting to use a scalpel rather than an ax, and to focus effort and resources first on prevention of the in silico and laboratory creation of high-consequence harms rather than control of such harms after-the-fact.

Below, we elaborate on how previously identified dual-use capabilities in the life sciences could inform tangible and testable components of biological AI model evaluations. Table 1 lists previously identified categories of dual-use research in the life sciences. We have generated examples of emerging AI “capabilities of concern” corresponding to each category. Of the examples listed, only one has been fully realized and scientifically validated—the optimization and generation of viral serotypes capable of evading immunity [55]. The design of molecules with increased toxicity has been explored computationally [56,57], and several other emerging AI capabilities of concern are under active development.

**Table 1:** Categories of dual-use research in the life sciences warranting risk assessment, as previously identified by scientists and US policymakers [30], and corresponding emerging AI capabilities. Note that the emerging AI-enabled capabilities constitute an illustrative, not exhaustive, list.

Experimental Outcomes Warranting Risk Assessment	Corresponding Emerging AI-enabled Capabilities of Concern
**Category 1 Research: Dual-Use Research of Concern**	
*Increase transmissibility of a pathogen within or between host species*	Design, or modeling of directed evolution towards, transmissibility characteristics of a pathogen through genome, protein, or other pathogen property alterations (while maintaining pathogen fitness).
	High-throughput screening and data generation methods for pathogen transmission traits, which may be used to create datasets for training AI models^1^.
*Increase the virulence of a pathogen or convey virulence to a non-pathogen*	Design, or modeling of directed evolution towards, virulence characteristics of a pathogen through genome, protein, or other pathogen property alterations (while maintaining pathogen fitness). This includes enhancing virulence, specifying delayed onset of virulence, and rendering nonpathogens or dormant pathogens virulent.
	High-throughput screening and data generation methods for pathogen virulence traits^1^.
*Increase the toxicity of a known toxin or produce a novel toxin*	Design of molecules, peptides or proteins with toxicity.
	High-throughput screening and data generation methods for molecule, peptide or protein toxicity^1^.
*Increase the stability of a pathogen or toxin in the environment, or increase the ability to disseminate a pathogen or toxin*	Modeling and design of stability and aerosolization characteristics of a pathogen or toxin in the environment, for example under specified temperature and humidity conditions.
	Modeling and design of resistance to physical countermeasures of a pathogen, such as filtration or disinfectants.
	High-throughput screening and data generation methods for evaluating environmental stability or aerosolization^1^.
	Prediction or design of structural features that constrain genetic changes, and thereby enable genomic engineering that resists evolutionary select pressures.
	Causal AI modeling of expected epidemiological spread (with and without mitigating interventions) based on pathogen genomic data.
*Alter the host range or tropism of a pathogen or toxin*	Design of genomes, genetic pathways or proteins that convert non-human animal pathogens into human pathogens.
	Design of genomes, genetic pathways or proteins that expand or target a pathogen host or tropism range.
	High-throughput screening methods for viral tropism traits, including host, tissue, and cellular tropism^1^.
*Decrease the ability for a human or veterinary pathogen or toxin to be detected using standard diagnostic or analytical methods*	Design of genetic sequences with high functional homology and no/low sequence homology which enable evasion of sequence-homology-based DNA synthesis screening methods, or sequence-based diagnostics.
*Increase resistance of a pathogen or toxin to clinical and/or veterinary prophylactic or therapeutic interventions*	Design of pathogen genomes, genetic pathways, or proteins that confer resistance to prophylactics or therapeutics.
*Alter a human or veterinary pathogen or toxin to disrupt the effectiveness of preexisting immunity, via immunization or natural infection, against the pathogen or toxin*	Design of pathogen genomes, genetic pathways, or proteins that enable immune escape. For example, optimizing viral vectors or generating viral serotypes that evade existing natural or vaccine-generated immunity.
*Enhance the susceptibility of a host population to a pathogen or toxin*	Design of pathogen genomes, genetic pathways or proteins that specify susceptibility of particular host populations, such as human ethnic, sex or age groups.
	Design of toxins which affect particular host populations, such as human ethnic, sex or age groups.
**Category 2 Research: Pathogens with Pandemic Potential**	
*Enhance transmissibility of the pathogen in humans*	Design, or modeling of directed evolution towards, transmissibility characteristics of a human pathogen through genome, protein, or other pathogen property alterations (while maintaining pathogen fitness).
	High-throughput screening and data generation methods for human pathogen transmission traits, which may be used to create datasets for training AI models^1^.
*Enhance the virulence of the pathogen in humans*	Design, or modeling of directed evolution towards, virulence characteristics of a human pathogen through genome, protein, or other pathogen property alterations (while maintaining pathogen fitness). This includes enhancing virulence, specifying delayed onset of virulence, and rendering nonpathogens or dormant pathogens virulent.
	High-throughput screening and data generation methods for human pathogen virulence traits^1^.
*Enhance the immune evasion of the pathogen in humans such as by modifying the pathogen to disrupt the effectiveness of pre-existing immunity via immunization or natural infection*	Design of pathogen genomes, genetic pathways, or proteins that enhance human immune escape. For example, optimizing viral vectors or generating viral serotypes that evade existing natural or vaccine-generated immunity in humans.
*Generate, use, reconstitute, or transfer an eradicated or potential pandemic pathogen (PEPP), or a previously identified pathogen with enhanced pandemic potential (PEPP)*	AI-enabled assistance or autonomous completion of step-by-step laboratory protocols for the de novo synthesis of pandemic pathogens. This includes the assembly of large DNA constructs and booting of synthetic genomes in cells.
	AI-enabled assistance or obfuscation of efforts to acquire, use or transfer PPPs or PEPPs, including the evasion of DNA synthesis screening.

^1^AI-enabled methods are being used to advance these capabilities; however, these capabilities may also be enabled via non-AI based methods.

## Next steps for biosecurity evaluations of biological AI models

The capabilities of concern we illustrate in Table 1 are generic. These high-level capabilities next need to be translated into targeted, standardized evaluations. The UK, US and EU governments are now working to create standardized evaluations [20,29,58]. We recommend governments and model developers establish these standardized evaluations such that they are pathogen-agnostic, and assess the capabilities described in Table 1. While all listed capabilities of concern in Table 1 warrant assessment, we recommend prioritizing those in Category 2, due to their potential to simplify, accelerate or enable biological work capable of causing novel human pandemic, animal panzootic, or plant pandemics, or other widespread environmental harm.

**Fig 1:**
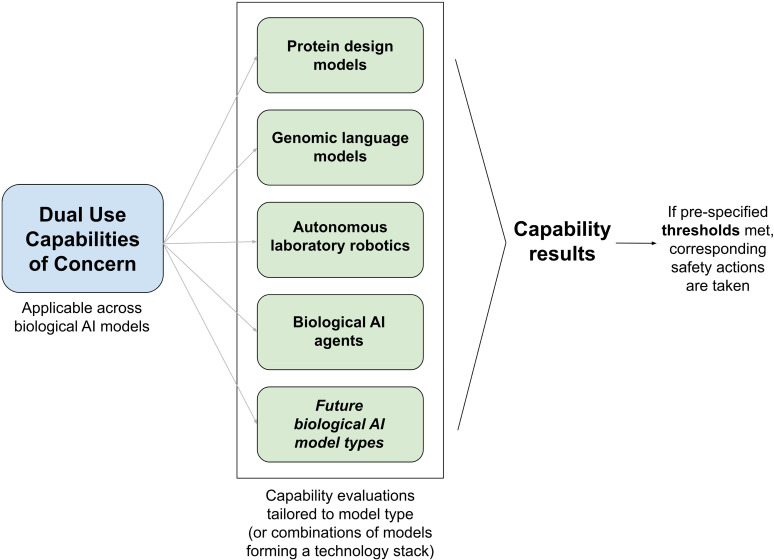
Biosecurity evaluation development should follow a distinct process. This figure is a simple schematic demonstrating that distinct evaluative methods will be needed for different types of biological AI models. Dual-use capabilities of concern should be translated into model type-specific evaluation methods that are linked to pre-specified thresholds and risk mitigation actions.

The specifics of evaluations will vary depending on the type and architecture of the AI model being evaluated (see Fig 1). Specific AI architectures, such as transformer-based LLMs, diffusion models, reinforcement learning agents, genomic foundation models and others, are likely to lend themselves to certain applications and thus different high-consequence capabilities. Most evaluation methods to date have been developed specifically for LLMs and pertain to human uplift; alternative approaches will be needed for biological AI models, which cannot be interrogated using LLM-specific methods. While an LLM evaluation may take the format of a natural language question bank [59], a biological AI model evaluation may take the format of other computational tasks. For example, biosecurity evaluation of the genomic foundation model Evo 2 involved repurposing the existing in silico performance benchmark ProteinGym [28,60].

Developing appropriate evaluations will require determining how to concretely measure these capabilities, otherwise, evaluations will risk being overly subjective and inconsistent. Accurate measurement is value-neutral; if measurements determine that the vast majority of biological AI models cannot meaningfully achieve these capabilities of concern, this would be extremely informative to the biosecurity community. One advantage of the aforementioned question and task-based approaches, versus evaluation methods that rely heavily on human judgement, is the potential for these methods to be standardized and measurable. At the same time, a degree of flexibility and fast iteration in the design and development of evaluations will be required, in order for methods to keep pace with AI developments.

Advancements in the above-described capabilities of concern are likely to correlate to some degree; further work is needed to assess this. For example, advancements in methods for high-throughput data generation (which themselves may be AI-enabled) are likely to lead to advancements in other model capabilities. As governments invest in data generation [61], data governance approaches should also be developed.

The need for wet-lab validation remains an ongoing challenge when assessing the performance of generative biological AI models; this challenge extends to safety evaluations as well. To determine the viability and function of a novel genetic sequence or protein, for example, one currently must create and test that novel biological design in vitro and/or in vivo. Such validation of dual-use biological AI models poses accident risks, which has prompted the US government to request the development of “peaceful proxy” evaluations [62]. An ideal proxy evaluation should accurately assess the desired capability of concern while minimizing potential accident risks during validation.

As mentioned above, a global consortium of biological AI model developers recently adopted the Responsible AI × Biodesign statement of community values and commitments [13]. Signatories committed to developing pre-release evaluations to assess potentially dangerous model capabilities, though these capabilities were not defined, and standards for how best to conduct rigorous evaluations have not yet been discussed. Current norms and incentives in academia push toward open release of code and model weights, which presents additional oversight challenges.

Some companies have stated that they have not publicly shared the content of their biosecurity evaluations due to information hazard concerns. To address this, policymakers should consider developing private, secure infrastructure amongst AI developers to share their biosecurity evaluations with one another and their respective governments.

Once completed, the results of evaluations for biological AI capabilities of concern should be linked with pre-specified thresholds and corresponding risk-mitigation actions (see Fig 1). Pre-specified thresholds refer to pre-set determinations regarding what degree of capability increase poses an unacceptable level of risk. For example, OpenAI’s Preparedness Framework defines low, medium, high and critical thresholds, with the critical threshold being breached when a “model enables an expert to develop a highly dangerous novel threat vector OR [a] model provides meaningfully improved assistance that enables anyone to be able to create a known CBRN threat OR [a] model can be connected to tools and equipment to complete the full engineering and/or synthesis cycle of a regulated or novel CBRN threat without human intervention” [63]. This language begins to move toward evaluation of biological capabilities, in particular capabilities relevant to accidental or deliberate misuse. Anthropic’s Responsible Scaling Policy and Claude 3 Model Card suggest that the company’s threshold is crossed when a model achieves “25% in accuracy on a set of advanced bioweapon-relevant questions … compared to using Google alone” [64,65]. This assessment appears to focus solely on bioweapons development, rather than specific capabilities, though Anthropic has not disclosed the exact content of these questions.

AI developers must be able to clearly identify when a model has reached a scientifically agreed-upon threshold of unacceptable risk. Furthermore, the suite of risk-mitigation actions that can be taken once capability thresholds are met needs to be explicated. AI risk mitigation measures will be distinct from those focused on mitigating wet-lab risk dual-use risks. Examples of prevention and mitigation strategies for biology AI models under consideration include: exclusion of concerning data from training [28], removing dangerous information from a model after the initial training has been completed via *machine unlearning* methods [59,66], restricting access to a model to specific users via APIs or other secure means, and/or subjecting models to governmental risk-benefit assessment. AI developers should disclose risk-mitigation requirements before training and testing relevant models to reassure the public that new AI models that pose serious biological risks will not be publicly released.

Given the speed of AI technological advances, assessments of real-world risk can no longer be expected to be static or unchanging over long periods of time, and developers and policymakers must regularly update their risk thresholds by drawing on the results of evaluations. Ultimately, the goal of biosecurity evaluations for biological AI models should be to provide targeted risk-reduction of high-consequence harms. Criteria for evaluations should be clear and standardized, allowing for beneficial research to easily proceed without undue impediment. We hope that these proposed categories of dual-use capabilities of concern can be used to help set standardized biosecurity evaluations for AI models. We encourage scientists, AI developers, and policymakers to create international standards and requirements for the development of safe and secure AI systems.
